# Development of the neonatal pain response variable set: a mixed methods consensus process

**DOI:** 10.1007/s00431-024-05559-7

**Published:** 2024-06-08

**Authors:** Nanxi Zhu, Bingjie Long, Xinling Zhan, Lanxin Zhang, Zechuan Wang, Lianhong Wang, Yi Huang, Juan Chen, Chi Huang, Lu Xiong, Zhenyan Fu, Renli Deng

**Affiliations:** 1 Department of Nursing, Affiliated Hospital of Zunyi Medical University, Zunyi, China; 2 Nursing School, Zunyi Medical University, Zunyi, China; 3 Department of Neonatology, Affiliated Hospital of Zunyi Medical University, Zunyi, China

**Keywords:** Neonate, Pain, Nursing assessment, Influencing factor, Assessment Indicator

## Abstract

While over 40 neonatal pain assessment scales have been published, owing to a lack of consensus and standardized metrics, there are more than 100 assessment indicators with varying descriptors and quality differences. This study aims to reach a consensus on optimal and comprehensive variables for neonatal pain assessment, leading to the development of a multidimensional neonatal pain response variable set. This study consisted of three phases: (1) A literature review was conducted to identify influencing factors and assessment indicators of neonatal pain response. (2) Panel meetings involving neonatal healthcare professionals evaluated and screened factors and indicators to develop an initial draft of the variable set. (3) Through two rounds of Delphi study achieved consensus, and determined the neonatal pain response variable set. Through a literature review and a panel meeting, the identified factors and indicators were categorized into contextual, physiological, and behavioral variables, forming an initial draft of the variable set. Sixteen professionals participated in two rounds of the Delphi study, with response rates exceeding 70%, and authority coefficients surpassing 0.7 in both rounds. The final iteration of the variable set includes 9 contextual variables, 2 physiological variables, and 5 behavioral variables.

*   Conclusion*: Neonatal pain response variable set developed in this study is scientific, comprehensive, and multidimensional, aligning with the characteristics of neonatal pain response and clinically applicable. The inclusion of contextual variables enhances the ability to confront the complexity of clinical environments and individual differences. It can provide a practical and theoretical basis for clinical research on neonatal pain assessment.

**Table:** 

**What is Known:** *• Neonatal pain assessment relies on scales used by healthcare professionals currently. But there is no “gold standard” for neonatal pain assessment.* *• While over 40 neonatal pain assessment scales have been published, owing to a lack of consensus and standardized metrics, there are more than 100 assessment indicators with varying descriptors and quality differences. Most of scales overlook the clinical environment complexity individual differences in pain responses, diminishing the accuracy and applicability.*	
**What is New:** *• In addition to the commonly used physiological and behavioral variables in the scales, we have incorporated contextual variables to better address the complexity of clinical environments and individual differences in pain responses.* *• Through an evidence-based approach, developed a neonatal pain response variable set comprising 9 contextual variables, 2 physiological variables, and 5 behavioral variables.*

**Supplementary Information:**

The online version contains supplementary material available at 10.1007/s00431-024-05559-7.

## Introduction

Pain has been designated by the World Health Organization(WHO) as the fifth vital sign [1]. Neonates exhibit central sensitization to repeated painful stimuli, which can adversely affect brain microstructure, lead to the production of stress hormones, impair cognitive and motor development, and contribute to behavioral issues, metabolic disorders, and cardiovascular stress [2]. Advances in neonatal care have improved the survival rates of premature and ill neonates. However, this progress has led to an increased number of invasive procedures that may induce pain in these vulnerable infants [3, 4]. Neonates exhibit a lower pain threshold (30 to 50% lower than that of adults) and reduced pain tolerance compared to older children, their experience of pain is often more intense and enduring [5, 6]. Optimal neonatal pain management requires valid non-verbal pain assessment, yet clinical methodology to assess neonatal pain is rudimentary [7]. Due to limitations in neonatal language expression, it fully depends on nurses to assess their pain through observation currently. The challenge for consistency increases when pain measures are subjective in nature [8].

Currently, there is no universally accepted “gold standard” for assessing neonatal pain, most clinical assessments rely on neonatal pain assessment scales. Over 40 such scales have been published [9], with distinct variations among which factors are included in the assessment. There are more than 100 evaluation indicators with varying descriptors utilized in the scales, lacking consensus and standardized metrics. General limitations with these scales have meant that clinicians and researchers have adapted and tweaked them without the necessary validation steps to make them more appropriate for use in their specific settings. This inconsistency makes it difficult to combine and synthesize evidence about how much pain infants are experiencing and whether interventions to prevent or alleviate pain are efficacious [10]. Simultaneously, it is decreasing the rate of neonatal pain assessment, posing challenges to ensure its accuracy. Globally, the rate of pain assessment in neonatal units ranges from a mere 6 to 50.3% [11–13]. Accurate pain assessment is crucial to ensuring the effectiveness and safety of pain management therapies in neonates during their stay in the Neonatal Intensive Care Unit (NICU). However, accurately measuring neonatal pain remains the greatest challenge in neonatal pain management [10, 14].

Most scales are too complicated and require live measurements and calculations (e.g., the Premature Infant Pain Profile scale requires the operator to calculate the percentage of decrease in SaO_2_ and of increase in heart rate in the first 30 s after the pain onset) that cannot be done in real-time and in particular by a single operator who is simultaneously performing the potentially painful procedure [15]. The clinical environment is complex, the passive state of newborns during hospitalization because of treatment methods or constraints may limit the sensitivity of certain behavioral or physiological indicators. Single-dimensional indicators (such as facial expressions, body movements, etc.) lack sufficient specificity to enable accurate pain measurement by nurses. Practicality and clinical utility are also significant when choosing an assessment tool for research or clinical use [8]. Moreover, pain responses exhibit individual differences, with demographic contextual factors contributing to explaining it. For example, preterm infants may lack the motor skills to express pain compared to full-term infants [16], and there are significant negative correlations between infants’ birth weight and parameters of pain severity [17]. But most of the scales ignore it.

In light of these challenges, this study conducted a systematic literature review to gather not only neonatal pain response influencing factors but also integrated multiple assessment items from various pain scales. Through panel meetings and the Delphi study, we selected the most representative and clinically practical indicators to develop a multidimensional neonatal pain response variable set, which aligns with the clinical characteristics of neonatal pain and ensures ease of pain observation and assessment. It is anticipated that this will facilitate future research on standardized tools and procedures for the clinical assessment of neonatal pain.

## Method

This study conducted a three-stage mixed-methods study to develop a set of neonatal pain response variables. Commencing with a literature review to extract influencing factors and assessment indicators of neonatal pain response. Subsequently, a panel meeting was conducted to screen and modify the gathered information, resulting in the initial draft of the neonatal pain response variable set. Finally, two rounds of the Delphi study were carried out to achieve consensus on the neonatal pain response variables.

### Literature review

A comprehensive systematic search up to December 2021 was conducted across WANFANG, CNKI, CBM, PubMed, Embase, Web of Science, and Cochrane. The search utilized combinations of terms/keywords such as “infant,” “newborn,” “child,” “neonate,” “baby,” “pain response,” “pain reaction,” and “factor,” considering both original research articles, guidelines, and consensus as study types. All records and data were managed using EndNote X9. Duplicate literature was eliminated, followed by screening of titles and abstracts followed by a full-text screening for eligibility was performed by 2 researchers independently, with any disagreements resolved by a third researcher or through group discussion. I dentifying relevant influencing factors and pain assessment indicators in neonatal pain research, ensuring that variables can best capture essential pain-related information.

### Panel meeting

To ensure both the clinical applicability and scientific validity of each variable assessment item, and to align with the clinical characteristics of neonatal pain responses, healthcare professionals specializing in neonatology, pediatric thoracic surgery, and pediatric internal medicine were recruited for an in-person panel meeting. Pertinent information regarding the meeting’s agenda was sent one week in advance. During the meeting, panelists provided opinions and feedback on the neonatal pain response variables and descriptors extracted from the literature review. It is advisable to have a panel of experts ranging from 10 to 15 members to ensure the quality of the meeting [18]. Post-meeting, the initial draft of the neonatal pain response variable set was generated for the subsequent Delphi study.

### Delphi study

Invited experts specializing in the field of pediatrics to participate in the consultation to gather and synthesize expert opinions and achieve consensus on the neonatal pain response variables, determine the final draft of neonatal pain response variables, and establish a variable set. Guided by Grime et al.’s suggestion of involving 5–20 participants [19], we invited 16 experts with a minimum of 10 years of experience in the field of pediatrics. All hold bachelor’s degrees or higher and possess senior academic titles of associate professor or above, ensuring broad professional representation.

The survey was conducted either face-to-face or through the online platform www.wjx.cn, with responses collected within one week of questionnaire distribution. The first round of questionnaires was designed based on the initial draft of neonatal pain response variables. The questionnaire comprised four main sections: (1) Introduction to the research content and significance of the topic; (2) General information about the experts; (3) Evaluation of pain response variables; and (4) Experts’ self-assessment of familiarity and judgment criteria for the consultation content. Expert panelists used a 5-point Likert scale (1 = strong disagreement, 3 = neither agreement nor disagreement, 5 = strong agreement) to rate the importance of each variable. An expert opinion column was provided for each variable, along with a blank column at the end of the consultation content for experts to offer additional opinions. Variables with an importance rating > 3.5 and a coefficient of variation ≤ 0.25 were considered for inclusion, following the criteria outlined by Hasson et al. [20]. The second round of questionnaires was created after modifying variable content based on expert opinions. Subsequent consultation rounds were conducted until a consensus was reached.

Data analysis was carried out using Excel 2016 and SPSS 29.0: (1) General information about experts: Descriptive statistics included mean and standard deviation for measurement data and frequency and percentage for count data. (2) Expert active coefficients: effective survey response rate was used. (3) Expert authority coefficient: expressed through the authority coefficient (Cr), derived as the average of the judgment coefficient (Ca) and the familiarity coefficient (Cs). Ca represented the basis for expert judgments on consultation content, while Cs reflected experts’ self-assessment of familiarity with the content. (4) Expert opinion consensus level: assessed using the coefficient of variation (CV) and Kendall's W coefficient.

## Result

### Literature review

**Fig. 1:**
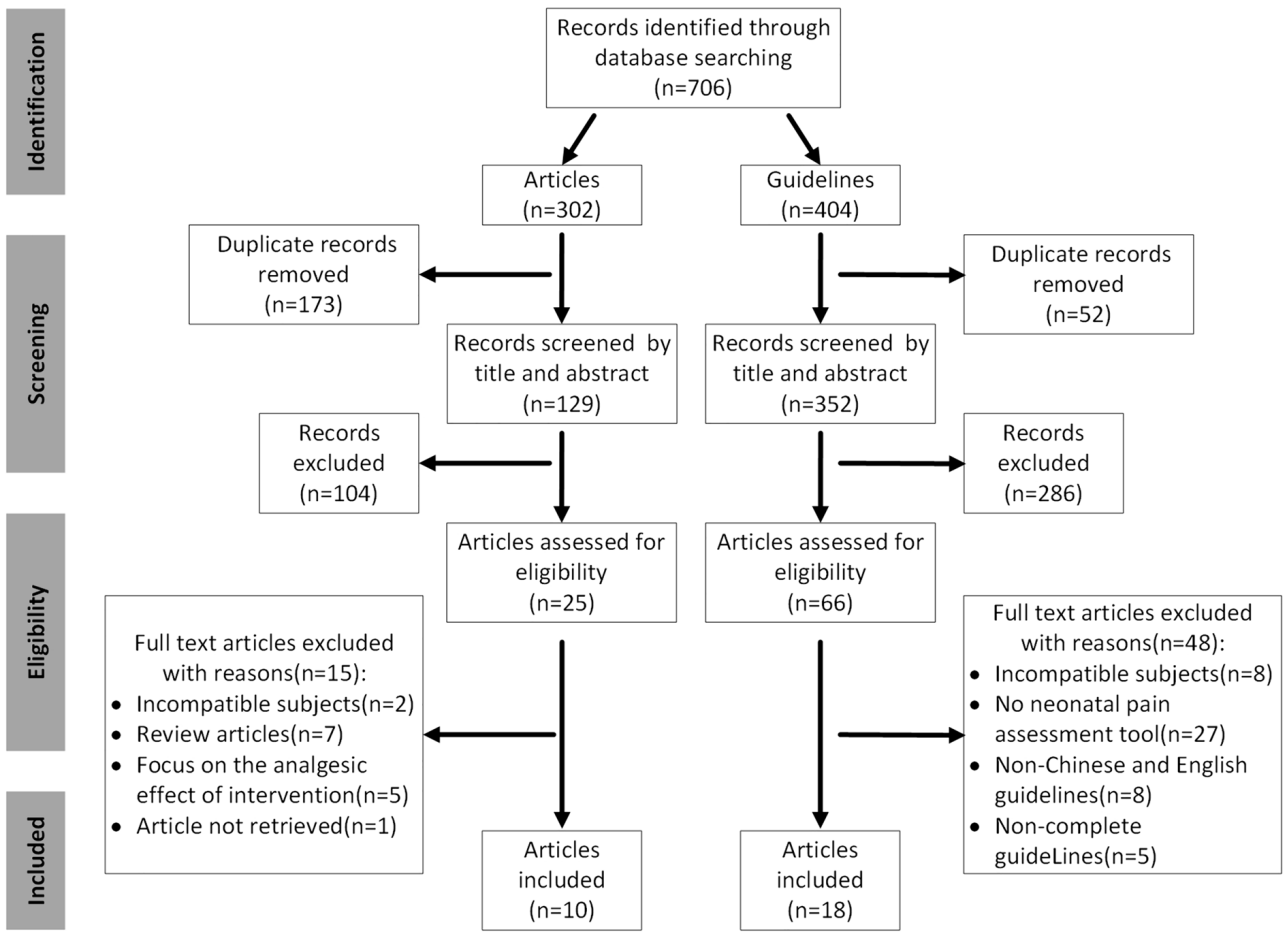
Flow diagram of the systematic literature search

The literature search yielded a total of 706 results, 10 articles and 18 guidelines met the inclusion criteria and underwent data extraction. Figure 1 illustrates the systematic flow of the literature search. From the 10 identified articles, 10 influencing factors of neonatal pain response were included. Among the 18 included guidelines, a total of 131 pain assessment tools were recommended. After removing duplicates, tools not applicable to neonates, and those not validated in neonatal populations, we ultimately selected 21 tools, from which we extracted a total of 123 assessment indicators. Through discussions and synthesis of similar indicators, three types of variables were classified: contextual, physiological, and behavioral, totaling 26 indicators. The extracted results of variables are presented in Table 1*.* The included articles, guidelines, and the 21 neonatal pain assessment scales recommended by the guidelines are provided in Appendix 1.

**Table 1:** The extracted results of influencing factors and assessment indicators

**Number**	**Type of variable**	**Extracted influencing factors/assessment indicators**
1	**Contextual variable**	Hospital days^a^
2		Sleeping/Wake state^a^
3		Mode of delivery^a^
4		Age^a^
5		Birth weight^a^
6		GA^a^
7		Procedure types^a^
8		Apgar (1 min)^a^
9		Apgar (5 min)^a^
10		Gender^a^
11		GA
12		Nurse's perception
13	**Physiological variable**	Requires O_2_ for Sat > 95
14		Oxygen required
15		SaO_2_ changes
16		SpO_2_ changes
17		Heart rate changes
18		Breathing changes
19		Blood pressure
20		Color
21	**Behavioral variable**	Baseline behavioral state
22		Sleep/Wake State
23		Alertness
24		Calmness/Agitation
25		Sleep pattern
26		Quality of sleep
27		Facial action
28		Crying
29		Duration of crying
30		Time to calm
31		Reacting to handling
32		Consolability
33		Breathing pattern
34		Body movements
35		Muscle tone
36		Posture of the trunk

^a^Extracted influencing factors

### Panel meeting

A total of 12 experts were included in this expert panel meeting. The experts' clinical areas of expertise encompassed neonatology, pediatric general thoracic surgery, and pediatric internal medicine, comprising both nurses and physicians. Among them, 8 held associate professor titles, and 4 held full professor titles. In terms of educational background, there was 1 expert with a bachelor’s degree, 9 with master’s degrees, and 2 with doctoral degrees. Their years of clinical experience ranged from 10 to 31 years.

In influencing factors, the factors “Sleep/Wake state” and “Gestational age (GA)” were eliminated due to repeated assessment indicators. The experts emphasized that assessment indicators and their descriptors should align with clinical reality, ensuring their broad applicability. Therefore, in assessment indicators, “Blood pressure changes”, “Alertness”, “Muscle tone” and “Body posture” were recommended for deletion. Similarly, some descriptors that posed challenges in a clinical setting were either removed or modified. Furthermore, experts recommended comparing “changes” to baseline values and using specific value change ranges for a more intuitive assessment. For instance, the description of “heart rate changes” was revised to indicate the frequency of increases or decreases compared to the baseline. Following summarizing the experts’ opinions, we further discussed and revised the relevant content, developing the first draft of the neonatal pain response variable set. The details of experts’ opinions and the first draft of the neonatal pain response variable set are presented in Appendix 2.

### Delphi study

Sixteen experts, consisting of nurses and physicians, from two general hospitals in Guizhou and Guangdong provinces of China, were invited from neonatology, neonatal intensive care units (NICUs), pediatric internal medicine, and pediatric general thoracic surgery departments. All these experts had over 10 years of clinical experience in pediatric-related fields. Among them, 3 had undergraduate degrees, 10 had master’s degrees, and 3 had doctoral degrees. Participants in both rounds of the survey remained consistent, and the effective survey response rate exceeded 70%, indicating high expert interest and attention. The authority coefficient (Cr), calculated as (Ca + Cs)/2, was 0.875 in the first round and 0.885 in the second round, both exceeding 0.7, signifying high expert authority and reliable consultation results.

In the first round, variables were screened using the criteria of importance assignment mean > 3.5 and coefficient of variation ≤ 0.25. Thirteen variables, including “nurse’s perception”, “requires O_2_ for Sat > 95”, and “SaO2 changes”, were deleted. “Duration of crying” was recommended to be retained with modified descriptors, specifying the duration as the time elapsed from the onset of crying to its cessation following pain stimulation. The details of experts’ opinions are presented in Appendix 2.

In the second round, responses, modifications, and explanations based on first-round comments were presented. Experts reached a consensus on all variables except “duration of crying”, which was suggested to be deleted due to potential interference factors affecting newborns’ crying times during procedures. The results of the two rounds of the Delphi study are shown in Tables 2 and 3.

**Table 2:** The survey results in round 1

**Factor/indicator**	**Mj**	**S**	**CV**	**Result**
**Hospital days**	4.25	0.68	0.16	Retain
**Mode of delivery**	3.56	0.73	0.21	Retain
**Age**	4.38	0.62	0.14	Retain
**Birth weight**	4.06	0.85	0.21	Retain
**Procedure types**	4.63	0.5	0.11	Retain
**Gender**	3.63	0.62	0.17	Retain
**Apgar (1 min)**	4.31	0.79	0.18	Retain
**Apgar (5 min)**	4.63	0.62	0.13	Retain
**GA**	4.69	0.46	0.1	Retain
**Nurse’s perception**	3.5	1.15	0.33	Delete
**Requires** **O**_**2**_ **for** **Sat** **>** **95**	3.25	1.06	0.33	Delete
**Oxygen requirement**	2.81	0.83	0.3	Delete
**SaO**_**2**_ **changes**	2.31	0.6	0.26	Delete
**SpO**_**2**_ **changes**	4.69	0.48	0.1	Retain
**Heart rate changes**	4.75	0.45	0.09	Retain
**Breathing changes**	3.31	1.01	0.31	Delete
**Color**	2.88	0.96	0.33	Delete
**Baseline behavioral state**	3.81	0.66	0.17	Retain
**Sleep/wake state**	3.5	1.03	0.29	Delete
**Calmness/Agitation**	3.38	1.09	0.32	Delete
**Sleep pattern**	2.19	0.75	0.34	Delete
**Quality of sleep**	2.5	0.82	0.33	Delete
**Facial action**	5	0	0	Retain
**Crying**	5	0	0	Retain
**Duration of crying**	4.06	0.57	0.14	Retain
**Time to calm**	2.94	0.57	0.19	Delete
**Reacting to handling**	3.19	1.05	0.33	Delete
**Consolability**	2.44	0.81	0.33	Delete
**Breathing pattern**	3.75	0.93	0.25	Retain
**Body movements**	4.63	0.5	0.11	Retain

Screening criteria: importance assignment mean (Mj) > 3.5, coefficient of variation (CV) ≤ 0.25

**Table 3:** The survey results in round 2

**Factor**	**Mj**	**S**	**CV**	**Result**
**Hospital days**	4	0.37	0.09	Retain
**Mode of delivery**	3.5	0.73	0.21	Retain
**Age**	4.25	0.45	0.11	Retain
**Birth weight**	3.88	0.62	0.16	Retain
**Procedure types**	4.69	0.48	0.1	Retain
**Gender**	3.75	0.58	0.15	Retain
**Apgar (1 min)**	4.69	0.6	0.13	Retain
**Apgar (5 min)**	4.81	0.4	0.08	Retain
**GA**	4.75	0.45	0.09	Retain
**SpO**_**2**_ **changes**	4.75	0.45	0.09	Retain
**Heart rate changes**	4.94	0.25	0.05	Retain
**Baseline behavioral state**	3.81	0.66	0.17	Retain
**Facial action**	5	0	0	Retain
**Crying**	5	0	0	Retain
**Duration of crying**	3.44	0.73	0.21	Delete
**Breathing pattern**	3.56	0.89	0.25	Retain
**Body movements**	4.88	0.34	0.07	Retain

Screening criteria: importance assignment mean (Mj) > 3.5, coefficient of variation (CV) ≤ 0.25

Following two rounds of the Delphi study and extensive expert discussions, a consensus was reached, resulting in the establishment of a neonatal pain response variable set. This set included 9 contextual variables, 2 physiological variables, and 5 behavioral variables, as presented in Table 4.

**Table 4:** The neonatal pain response variable set

**Type of variable**	**Variable**	**Descriptor**
**Contextual variable**	Hospital days	/
	Mode of delivery	/
	Age	/
	Birth weight	/
	Procedure types	/
	Gender	/
	Apgar (1 min)	/
	Apgar (5 min)	/
	GA	/
**Physiological variable**	SpO_2_ changes	Change from the baseline: baseline________
		• 0–2;
		• 3–5;
		• 6–8;
		• > 8 or increasing in O_2_ requirement
	Heart rate changes	Change from the baseline: baseline________
		• Normal: heart rate decreases/increases 0–5 beats from baseline;
		• Slight change: heart rate decreases/increases 6–20 beats from baseline;
		• Clear change: heart rate decreases/increases > 20 beats from baseline
**Behavioral variable**	Baseline behavioral state	• Active and Awake;
		• Quiet and Awake;
		• Active and Asleep;
		• Quiet and Asleep
	Facial action	• Facial muscles fully relaxed, relaxed open mouth;
		• Normal facial tension;
		• Intermittent eye squeeze and brow furrow;
		• Continuous eye squeeze and brow furrow;
		• Facial muscles contorted and grimacing (eye squeeze, brow furrow, open mouth, nasal-labial lines)
	Crying	• No cry (quiet, not crying);
		• Whimper (mild moaning, intermittent);
		• vigorous crying (loud scream, shrill, continuous)
	Breathing pattern	• Relaxed (usual pattern for this infant);
		• Change in breathing (irregular, faster than usual, gagging, breath holding)
	Body movements	• No movement;
		• Up to three arm and/or leg movements;
		• More than three arm and/or leg movements

## Discussion

In this study, we focus on the characteristics of neonatal pain response, through a comprehensive data collection and meticulous screening process, involving a literature review, panel meetings, and a Delphi study, we aim to develop a neonatal pain response variable set, which serves as a crucial theoretical foundation for future research in the field of neonatal pain.

In its revised definition of pain in 2020, the International Association for the Study of Pain (IASP) emphasized the significance of respecting a person’s report of pain as a valid experience, the inability to communicate does not negate the possibility that a human or a non-human animal experiences pain [21]. Due to limited verbal communication, the primary mode of assessing neonatal pain responses is through observation. Nevertheless, the absence of a “gold standard” results in not all assessment indicators from existing scales being suitable for clinical practice, and the influence of situational factors, such as demographic contextual factors, on neonatal pain responses is frequently disregarded.

From a neurological standpoint, no individual behavioral or physiological component is adequate in alerting to the presence of pain responses that occur on a cortical level [22]. Our constructed variable set aligns with recommendations from the previous studies and guidelines [23, 24], emphasizing the importance of a multidimensional assessment approach that considers both behavioral and physiological responses. It aims to overcome the limitations of single-dimensional assessments, which often fail to adapt to the complexity of clinical environments and capture the sometimes-subtle pain behaviors exhibited by neonates. Furthermore, our variable set also incorporates contextual variables to address individual differences in neonatal pain, providing a comprehensive assessment framework composed of three key dimensions: behavioral, physiological, and contextual.

To enhance discrimination, we selected descriptors with improved discriminatory capabilities. Upon analyzing and comparing each variable’s descriptors, we considered the appropriateness of their scope and the clarity of their grade divisions. In the behavioral variables, the assessment of facial expressions expanded to include five dimensions: “Facial muscles fully relaxed, relaxed open mouth”, “Normal facial tension”, “Intermittent eye squeeze and brow furrow”, “Continuous eye squeeze and brow furrow”, and “Facial muscles contorted and grimacing”. These descriptions offer greater clarity and understanding compared to existing assessment scales, with well-defined boundaries closely aligning with neonatal pain-related facial expressions. For descriptors related to “crying” and “breathing pattern”, we used the contents of the NIPS scale, incorporating dimensions like “no cry”, “whimper”, and “vigorous crying” for crying assessment, and “relaxed” and “change in breathing” for breathing patterns. While some scales previously mentioned assessing “extremity movement” via finger and toe movements, we recognized the impracticality of these observations due to medical interventions or protective measures. Therefore, we opted to assess the frequency of arm and/or leg movements, categorizing them as “no movement”, “up to three arm and/or leg movements”, and “more than three arm and/or leg movements”, which enhances assessment applicability, efficiency, and accuracy.

Concerning physiological variables, we defined “SpO_2_” in terms of its deviation from baseline values, categorizing it as a decrease in Oxygen Saturation: “0 ~ 2”, “3 ~ 5”, “6 ~ 8”, or “ > 8”, or indicating an “increased oxygen requirement”. This definition deviates from the BPSN scale’s “decrease ≤ 1.9%”, “decrease 2 ~ 2.9%”, “decrease 3 ~ 4.9%”, and “decrease ≥ 5%”. Experts noted that a broader fluctuation range better reflects actual SpO_2_ changes in clinical newborns. Furthermore, our clinical observations revealed that SpO_2_ levels do not necessarily decrease during painful episodes, thus our descriptors extend beyond mere increases or decreases. For assessing “heart rate”, we employed a straightforward definition based on specific numerical changes compared to baseline values, categorizing them as “0 ~ 5 times”, “6 ~ 20 times”, or “ > 20 times”. It offers greater intuitiveness and practicality compared to percentage-based calculations used in some assessment tools.

The limitations of this study encompass the included literature’s language restriction to 2 languages, did not explicitly consider the potential influence of regional factors on neonatal pain response, and the articles primarily focused on hospitalized newborns, potentially overlooking research on healthy newborns, which could affect the comprehensiveness of included literature and the factors related to neonatal pain extracted in this study. Our next objective is to construct a neonatal pain response model, that serves as a valuable tool for healthcare practitioners. This model aims to empower healthcare professionals in assessing the significance of each pain-related variable.

## Conclusion

In this study, we developed a neonatal pain response variable set through a literature review, panel meeting, and Delphi study. This set includes three dimensions: contextual variables, physiological variables, and behavioral variables, totaling sixteen variables, establishing a structured framework for neonatal pain assessment and management.

### Supplementary Information

Below is the link to the electronic supplementary material.

Supplementary file1 (DOCX 99 KB)

Supplementary file2 (DOCX 84 KB)

## Data Availability

The data that support the findings of this study are available from the corresponding author, upon reasonable request.

## Abbreviations

NICU: Neonatal Intensive Care Unit
WHO: World Health Organization
