# Supplementary material for: Development of the neonatal pain response variable set: a mixed methods consensus process
Source: Eur J Pediatr. 2024 Jun 8;183(9):3719–26. doi: 10.1007/s00431-024-05559-7 (PMC11322254; doi:10.1007/s00431-024-05559-7)
Supplement: Supplementary file 1 — Supplementary file1 (DOCX 99 KB) [file 431_2024_5559_MOESM1_ESM.docx]

| **Table 1 Characteristics of Included Articles(n=10)** | | | |
| --- | --- | --- | --- |
| **Articles** | **Publication Date** | **Study design** | **Influencing factors** |
| **Study on the Incidence and Influencing Factors of Procedural Pain in Neonatal Intensive Care Unit Patients[1]** | 2017 | Descriptive study | Type of pain experience, GA, Age, Birth weight, Hospital days, Pain duration, Frequency of Pain Experience, sleeping/wake situation |
| **Does mode of delivery influence the pain response of infants at their first vaccination?[2]** | 2009 | Descriptive study | Mode of delivery |
| **Effects of age, gender and holding on pain response during infant immunization[3]** | 2004 | Randomized controlled study | Age, Gender, Lying or Holding |
| **Factors associated with infant pain severity undergoing immunization injections[4]** | 2018 | Observational descriptive study | Gender, Caregiver attendance, The experience of early nociceptive stimuli (circumcision), Mode of delivery, Weight (birth and current) |
| **Clinical Observation of Factors Influencing Pain in Neonates[5]** | 2009 | Observational descriptive study | Gender, GA, Birth weight, Mode of delivery, Age, Type of pain experience |
| **Developmental changes in response to heelstick in preterm infants: a prospective cohort study[6]** | 1996 | Prospective cohort study | Age, Weight, Days since birth, With assisted ventilation, Severity of illness |

Continued

| **The behavioral pain response to heelstick in preterm neonates studied longitudinally: description, development, determinants, and components[7]** | 2009 | Longitudinal study | Age, Gender, Race, Post Menstrual Age, Birthweight, Apgar at 1 min, Apgar at 5 min, Length of stay, Sepsis during hospital stay, Mechanical ventilation |
| --- | --- | --- | --- |
| **A prospective study of pain experience in a neonatal intensive care unit of China[8]** | 2012 | Prospective Study | GA, Birth weight, Sex, Labor, 1-min APGAR, 5-min APGAR, Procedure Types, Hospitalization, Ventilation, Other respiratory supports, |
| **Are there developmentally distinct motor indicators of pain in preterm infants?[9]** | 2003 | Observational descriptive study | Sex, GA at birth, Birthweight, Apgar (1 min), Score for Neonatal Acute Physiology-II, Neonatal Medical Index, |
| **Contextual factors associated with pain response of preterm infants to heel-stick procedures[10]** | 2013 | Descriptive study | GA, Gender, Continuous positive airway pressure, Mechanical ventilation, Disease, Respiratory arrest, PMA, Apgar (1 min), Apgar (5 min), Weight, Duration of HS procedure, Number of painful procedures |

| **Table 2 Characteristics of Included Guidelines (n=18)** | | | | |
| --- | --- | --- | --- | --- |
| **Guideline** | **Publication Date** | **Country** | **Organization / Author** | **Recommended Pain Assessment Tools** |
| **Expert Consensus on Pain Management and Sedation Therapy in Pediatric Intensive Care Unit (2013 Edition)[11]** | 2014 | China | The Subspecialty Group of Emergency Medicine, the Society of Pediatrics, Chinese Medical Association & The Subspecialty Group of Pediatrics, the Society of Emergency Medicine, Chinese Medical Association & The Society of Pediatric Critical Care, Chinese Medical Doctor Association | CRIES, Comfort-B, NRS, VAS, FLACC, FPS, CHEOPS |
| **Clinical Management of Procedural Pain in Neonates: Introduction to the Latest Policy Statement on Prevention and Management of Procedural Pain in Neonates by the American Academy of Pediatrics[12]** | 2017 | China | Juan Fan, Maojun Li, Qing Wu, Changhui Chen | NFCS, PIPP, N-PASS, BIIP, NIAPAS |
| **Expert Consensus on Neonatal Pain Assessment and Analgesic Management (2020 Edition)[13]** | 2020 | China | Neonatology Physicians Branch of the Chinese Medical Doctor Association & Editorial Committee of Contemporary Pediatrics Journal of China | PIPP, PIPP-R, N-PASS, NIPS, CRIES, NFCS, NIAPAS, EDIN |
| **Expert Consensus on Pain Management and Sedation Therapy in Pediatric Intensive Care Unit in China (2018 Edition)[14]** | 2018 | China | Emergency Medicine Group, Pediatric Branch, Chinese Pediatric Society, Chinese Medical Association & Pediatric Division, Emergency Medicine Branch, Chinese Society of Emergency Medicine, Chinese Medical Association & Pediatric Intensive Care Physicians Branch, Chinese Physicians Association | CRIES, Comfort, NRS, VAS, FLACC, FPS, CHEOPS |

Continued

| **Clinical recommendations for pain, sedation, withdrawal and delirium assessment in critically ill infants and children: an ESPNIC position statement for healthcare professionals[15]** | 2016 | The Netherlands | Julia Harris, Anne-Sylvie Ramelet, Monique van Dijk, Pavla Pokorna, Joke Wielenga, Lyvonne Tume, Dick Tibboel, Erwin Ista | PIPP, PIPP-R, N-PASS, Comfort-neo, Comfort, Comfort-B, MAPS, FLACC |
| --- | --- | --- | --- | --- |
| **Clinicians' quick reference guide to acute pain management in infants, children, and adolescents: operative and medical procedures. Pain Management Guideline Panel. Agency for Health Care Policy and Research, US Department of Health and Human Services[16]** | 1992 | USA | Healthcare Policy Research Institute of the United States Department of Health | Poker Chip Tool, The Word-Graphic Rating Scale |
| **Consensus guidelines on sedation and analgesia in critically ill children[17]** | 2006 | UK | United Kingdom Paediatric Intensive Care Society Sedation & Analgesia and Neuromuscular Blockade Working Group | Comfort, VAS, NRS |
| **Consensus statement for the prevention and management of pain in the newborn[18]** | 2001 | USA | K. J. S. Anand, MBBS, Dphil & International Evidence-Based Group for Neonatal Pain | Comfort, PIPP, CRIES, NFCS, CHEOPS, FLACC |
| **Good practice in postoperative and procedural pain management, 2nd edition[19]** | 2012 | UK | Association of Paediatric Anaesthetists of Great Britain and Ireland | FRS-R, VAS, NRS, Wong and Baker FACES Pain Scale, Visual analogue* and numerical rating scales, Pieces of Hurt Tool, MSPCT, PPPM, NCCPC-R, PPP |

Continued

| **Guideline statement: management of procedure-related pain in neonates[20]** | 2006 | Australia | Paediatrics & Child Health Division, The Royal Australasian College of Physicians | PIPP, NIPS, NFCS, CRIES |
| --- | --- | --- | --- | --- |
| **Japanese clinical guidelines for chronic pain in children and adolescents[21]** | 2012 | Japan | Task Force of clinical practice guidelines for chronic pain in children and adolescents, Japanese Society of Psychosomatic Pediatrics | VAS, CHEOPS |
| **Management of postoperative pain: A clinical practice guideline from the American pain society, the American society of regional anesthesia and pain medicine, and the American society of anesthesiologists' committee on regional anesthesia, executive committee, and administrative council[22]** | 2016 | USA | American Pain Society & American Society of Regional Anesthesia and Pain Medicine & Committee on Regional Anesthesia, Executive Committee, and Administrative Committee of the American Society of Anesthesiologists | NRS, VRS, VAS, FRS, Pain Thermometer |
| **Pain assessment[23]** | 2008 | UK | Association of Paediatric Anaesthetists of Great Britain and Ireland | PIPP, CRIES, NFCS, Comfort, Wong and Baker FACES Pain Scale, FPS-R, Visual analogue* and numerical rating scales, Pieces of Hurt Tool, MSPCT, FLACC, CHEOPS, PPPM, NCCPC-R, PPP, Revised FLACC |

Continued

| **Pain assessment in the nonverbal patient: position statement with clinical practice recommendations[24]** | 2006 | USA | American Society for Pain Management Nursing | CHIPPS, Comfort, CRIES, DSVNI, PIPP, RIPS, UWCH, CHEOPS, FLACC, DEGR Scale |
| --- | --- | --- | --- | --- |
| **Prevention and management of pain in the neonate: an update[25]** | 2006 | USA | American Academy of Pediatrics Committee on Fetus and Newborn & American Academy of Pediatrics Section on Surgery & Canadian Paediatric Society Fetus and Newborn Committee | PIPP, CRIES, NIPS, N-PASS, NFCS, PAT, SUN, EDIN, BPSN |
| **Prevention and Management of Procedural Pain in the Neonate: An Update[26]** | 2016 | USA | Committee On Fetus And Newborn And Section On Anesthesiology And Pain Medicine | NFCS, PIPP, N-PASS, BIIP, DAN, PIPP-R, FANS, NIPS, Comfort-neo, COVERS Neonatal pain scale, PAIN, PAT, SUN, EDIN, BPSN |
| **Reducing the pain of childhood vaccination: an evidence-based clinical practice guideline[27]** | 2010 | Canada | Anna Taddio; Mary Appleton; Robert Bortolussi; et al. | MBPS, FLACC, FPS-R, NRS, Poker Chip Tool |
| **Turkish Neonatal Society guideline on the neonatal pain and its management[28]** | 2018 | Turkey | Turkish Society of Neonatology | PIPP, CRIES, NIPS, N-PASS, NFCS, PAT, SUN, EDIN, BPSN, Comfort |

| **Table 3 Recommended neonatal pain assessment scales** | | | |
| --- | --- | --- | --- |
|  | Neonatal Pain Assessment Scales | Participant | Reliability and validity |
| 1 | CRIES[29] | 32 to 60 weeks gestation age | Validity with OPS: 0.73 |
|  |  |  | Discriminant validity：YES |
|  |  |  | Inter-rater reliability: 0.72 |
| 2 | COMFORTneo[30] | 24.6 to 42.6 weeks gestation age | Inter-rater reliability: 0.79 |
|  |  |  | Internal consistency: 0.84-0.88 |
|  |  |  | Concurrent validity with NRS: 0.54 - 0.83 |
|  |  |  | Sensitivity to Change: Yes |
| 3 | NFCS[31] | Premature infants | Internal consistency: 0.872 |
|  |  |  | Inter-rater consistency: 0.954 |
|  |  |  | Discriminant validity: Yes |
|  |  |  | Concurrent validity and convergent validity |
|  |  |  | Agreement with NIPS: 95% |
| 4 | PIPP-R[32] | ≥26 weeks gestation age | Content validity: Yes |
|  |  |  | Construct validity: Yes |
|  |  |  | Inter-observer consistency: 0.944–1.000 |
|  |  |  | Sensitivity: 91.5%, Selectivity: 88.5% |
| 5 | N-PASS[33] | 23 to 40 weeks gestation age | Interrater reliability: 0.85-0.95 |
|  |  |  | Internal consistency: Cronbach’s α of 0.82-0.87 |
|  |  |  | Convergent validity with PIPP: 0.61-0.83 |
|  |  |  | Construct validity: Yes |

Continued

| 6 | BIIP[34] | 24 to 31 weeks gestational age | Internal consistency: 0.82 |
| --- | --- | --- | --- |
|  |  |  | Inter-rater reliability: 0.80-0.92 |
|  |  |  | Construct validity: YES |
|  |  |  | Concurrent validity with NIPS: 0.28-0.64 |
| 7 | NIAPAS[35] | 23 to 42 weeks gestation age | Construct and content validity: YES |
|  |  |  | Concurrent validity with NIPS: 0.873 |
|  |  |  | Inter-rater reliability: 0.991-0.997 |
|  |  |  | Intra-rater reliability: 0.992-1.00 |
|  |  |  | Internal consistency: 0.723 |
| 8 | NIPS[36] | 26 to 47 weeks gestation age | Inter-rater reliability: 0.92-0.97 |
|  |  |  | Internal consistency: 0.87-0.95 |
|  |  |  | Concurrent validity: 0.53-0.84 |
| 9 | EDIN[37] | 26 to 36 weeks gestation age | Inter-rater reliability: 0.59-0.74 |
|  |  |  | Internal consistency: 0.86-0.94 |
|  |  |  | Construct validity: YES |
| 10 | MBPS[38] | 2 to 6 months | Internal consistency: 0.83-0.94 |
|  |  |  | Inter-rater reliability: 0.90-0.94 |
|  |  |  | Construct validity: YES |
| 11 | MAPS[39] | 0 to 31 months | Internal consistency: 0.68 |
|  |  |  | Interrater reliability: 0.68-0.91 |
|  |  |  | Content, concurrent, convergent validity: Yes |

Continued

| 12 | BPSN[40] | 27 and 6 days to 41 weeks and 1 day gestation age | Internal consistency: 0.86-0.97 |
| --- | --- | --- | --- |
|  |  |  | Interrater reliability: 0.98-0.99. |
|  |  |  | Concurrent and convergent validity with VAS and PIPP: 0.86-0.91 |
|  |  |  |  |
|  |  |  | Construct validity: Yes |
| 13 | CHIPPS[41] | Newborns, infants, and young children | Interrater reliability: 0.93 |
|  |  |  | Internal consistency: 0.96 |
|  |  |  | Content and construct validity: Yes |
|  |  |  | Specificity and sensitivity: Yes |
| 14 | COVERS Neonatal pain scale[42] | All gestational ages | Internal Consistency: 0.74-0.78 |
|  |  |  | Inter-rater Reliability: 0.80-0.82 |
|  |  |  | Correlation with modified PAT:0.74-0.81 |
| 15 | DAN[43] | 25 to 41 weeks gestation age | Internal consistency: 0.88 |
|  |  |  | Inter-rater agreement: Krippendorff R test of 91.2 |
|  |  |  | Specificity and sensitivity: Yes |
| 16 | FANS[44] | 24 to 40 weeks gestation age | Internal consistency: Cronbach’s α of 0.72 |
|  |  |  | Inter-rater agreement: 0.92 |
|  |  |  | Correlation with DAN: 0.76-0.93 |
| 17 | PAT[45] | 27 to 40 weeks gestation age | Interrater reliability: 0.85 |
|  |  |  | Face, construct validity: Yes |
|  |  |  | Correlation with CRlES: 0.76 |
| 18 | PAIN[46] | 26 to 47 weeks gestation age | Correlation with NIPS: 0.93 |
|  |  |  | Construct and criterion validity: Yes |

Continued

| 19 | RIPS[47] | Infant and children | Inter-rater agreement: 0.87 |
| --- | --- | --- | --- |
|  |  |  | Discrimination validity: Yes |
|  |  |  | Sensitivity: 0.23, specificity: 0.90 |
| 20 | SUN[48] | 24 to 40 weeks gestation age | Construct validity: Yes |
|  |  |  | Coefficient of Variation: 33% ± 8% |
|  |  |  | Content and discriminant validity: Yes |
| 21 | UWCH[49] | 0.08 to 2.5 years | Interrater reliability: 0.92 |
|  |  |  | Internal consistency: 0.93 |
|  |  |  | Content, construct, and criterion validity: Yes |

1. Yajing Wang (2017) A Study on the Incidence and Influencing Factors of Procedural Pain in Neonatal Intensive Care Unit Patients. Master's Thesis, Peking Union Medical College.

2. De Buck FA, Karel ; Deprest, Jan ; Van De Velde, Marc (2009) Does mode of delivery influence the pain response of infants at their first vaccination ? European Journal of Anaesthesiology 26: 144

3. Ipp M, Taddio A, Goldbach M, Ben David S, Stevens B, Koren G (2004) Effects of age, gender and holding on pain response during infant immunization. The Canadian journal of clinical pharmacology = Journal canadien de pharmacologie clinique 11:e2-7

4. Kassab M, Hamadneh S, Nuseir K, B AL, Hamadneh J (2018) Factors Associated With Infant Pain Severity Undergoing Immunization Injections. Journal of pediatric nursing 42:e85-e90

5. Yunli Huang, Yongqing Ye, Dongming Huang, Qiaozhen Wu, Yuqi Shi, Weiqiong Wang, Shuying Liang, Chaoxia Chen (2009) Clinical Observation on Factors Influencing Neonatal Pain. Chinese Journal of Nursing 44:709-711.

6. Johnston CC, Stevens B, Yang F, Horton L (1996) Developmental changes in response to heelstick in preterm infants: a prospective cohort study. Developmental medicine and child neurology 38:438-445

7. Williams AL, Khattak AZ, Garza CN, Lasky RE (2009) The behavioral pain response to heelstick in preterm neonates studied longitudinally: description, development, determinants, and components. Early Hum Dev 85:369-374

8. Chen M, Shi X, Chen Y, Cao Z, Cheng R, Xu Y, Liu L, Li X (2012) A prospective study of pain experience in a neonatal intensive care unit of China. Clin J Pain 28:700-704

9. Morison SJ, Holsti L, Grunau RE, Whitfield MF, Oberlander TF, Chan HW, Williams L (2003) Are there developmentally distinct motor indicators of pain in preterm infants? Early Hum Dev 72:131-146

10. Sellam G, Engberg S, Denhaerynck K, Craig KD, Cignacco EL (2013) Contextual factors associated with pain response of preterm infants to heel-stick procedures. Eur J Pain 17:255-263

11. Chinese Pediatric Society Emergency Medicine Group, Chinese Society of Emergency Medicine Pediatric Group, Pediatric Specialty Committee of Chinese Physician Association Intensive Care Physicians Branch (2014) Expert Consensus on Analgesia and Sedation Therapy in Pediatric Intensive Care Unit (2013 Edition). Chinese Journal of Pediatrics 52:189-193

12. Juan Fan, Maojun Li, Qing Wu, Changhui Chen (2017) Clinical Management of Procedural Pain in Neonates: Introduction to the Latest Policy Statement of the American Academy of Pediatrics on Prevention and Management of Procedural Pain in Neonates. Chinese Journal of Pediatric Emergency Medicine 24

13. Zhichun Feng, Rui Cheng, Yang Yang, Yuan Shi (2020) Expert Consensus on Neonatal Pain Assessment and Analgesic Management (2020 Edition). Chinese Journal of Contemporary Pediatrics 22 923-930

14. Chinese Pediatric Society Emergency Medicine Group, Chinese Society of Emergency Medicine Pediatric Group, Chinese Physician Association Pediatric Intensive Care Physician Branch (2019) Expert Consensus on Analgesia and Sedation Therapy in Chinese Pediatric Intensive Care Units (2018 Edition). Chinese Journal of Pediatrics 57:324-330

15. Harris J, Ramelet AS, van Dijk M, Pokorna P, Wielenga J, Tume L, Tibboel D, Ista E (2016) Clinical recommendations for pain, sedation, withdrawal and delirium assessment in critically ill infants and children: an ESPNIC position statement for healthcare professionals. Intensive care medicine 42:972-986

16. Panel PMG (1992) Clinicians' quick reference guide to acute pain management in infants, children, and adolescents: operative and medical procedures. J Pain Symptom Manage 7:229-242

17. Playfor S, Jenkins I, Boyles C, Choonara I, Davies G, Haywood T, Hinson G, Mayer A, Morton N, Ralph T, Wolf A (2006) Consensus guidelines on sedation and analgesia in critically ill children. Intensive care medicine 32:1125-1136

18. Anand KJ (2001) Consensus statement for the prevention and management of pain in the newborn. Archives of pediatrics & adolescent medicine 155:173-180

19. Ireland AoPAoGBa (2012) Good practice in postoperative and procedural pain management, 2nd edition. Paediatr Anaesth 22 Suppl 1:1-79

20. Division PCH, Physicians TRACo (2006) Guideline statement: management of procedure-related pain in neonates. Journal of paediatrics and child health 42 Suppl 1:S31-39

21. Ishizaki Y, Yasujima H, Takenaka Y, Shimada A, Murakami K, Fukai Y, Inouwe N, Oka T, Maru M, Wakako R, Shirakawa M, Fujita M, Fujii Y, Uchida Y, Ogimi Y, Kambara Y, Nagai A, Nakao R, Tanaka H (2012) Japanese clinical guidelines for chronic pain in children and adolescents. Pediatrics international : official journal of the Japan Pediatric Society 54:1-7

22. Chou R, Gordon DB, de Leon-Casasola OA, Rosenberg JM, Bickler S, Brennan T, Carter T, Cassidy CL, Chittenden EH, Degenhardt E, Griffith S, Manworren R, McCarberg B, Montgomery R, Murphy J, Perkal MF, Suresh S, Sluka K, Strassels S, Thirlby R, Viscusi E, Walco GA, Warner L, Weisman SJ, Wu CL (2016) Management of Postoperative Pain: A Clinical Practice Guideline From the American Pain Society, the American Society of Regional Anesthesia and Pain Medicine, and the American Society of Anesthesiologists' Committee on Regional Anesthesia, Executive Committee, and Administrative Council. The journal of pain 17:131-157

23. Howard R, Carter B, Curry J, Morton N, Rivett K, Rose M, Tyrrell J, Walker S, Williams G (2008) Pain assessment. Paediatr Anaesth 18 Suppl 1:14-18

24. Herr K, Coyne PJ, Key T, Manworren R, McCaffery M, Merkel S, Pelosi-Kelly J, Wild L (2006) Pain assessment in the nonverbal patient: position statement with clinical practice recommendations. Pain Manag Nurs 7:44-52

25. Batton DG, Barrington KJ, Wallman C (2006) Prevention and management of pain in the neonate: an update. Pediatrics 118:2231-2241

26. MEDICINE COFANaSOAAP (2016) Prevention and Management of Procedural Pain in the Neonate: An Update. Pediatrics 137:e20154271

27. Taddio A, Appleton M, Bortolussi R, Chambers C, Dubey V, Halperin S, Hanrahan A, Ipp M, Lockett D, MacDonald N, Midmer D, Mousmanis P, Palda V, Pielak K, Riddell RP, Rieder M, Scott J, Shah V (2010) Reducing the pain of childhood vaccination: an evidence-based clinical practice guideline. CMAJ : Canadian Medical Association journal = journal de l'Association medicale canadienne 182:E843-855

28. Yiğit Ş, Ecevit A, Köroğlu Ö A (2018) Turkish Neonatal Society guideline on the neonatal pain and its management. Turk pediatri arsivi 53:S161-s171

29. Krechel SW, Bildner J (1995) CRIES: a new neonatal postoperative pain measurement score. Initial testing of validity and reliability. Paediatr Anaesth 5:53-61

30. van Dijk M, Roofthooft DW, Anand KJ, Guldemond F, de Graaf J, Simons S, de Jager Y, van Goudoever JB, Tibboel D (2009) Taking up the challenge of measuring prolonged pain in (premature) neonates: the COMFORTneo scale seems promising. Clin J Pain 25:607-616

31. Xie W, Wang X, Huang R, Chen Y, Guo X (2021) Assessment of four pain scales for evaluating procedural pain in premature infants undergoing heel blood collection. Pediatr Res 89:1724-1731

32. Taplak A, Bayat M (2019) Psychometric Testing of the Turkish Version of the Premature Infant Pain Profile Revised-PIPP-R. Journal of pediatric nursing 48:e49-e55

33. Hummel P, Puchalski M, Creech SD, Weiss MG (2008) Clinical reliability and validity of the N-PASS: neonatal pain, agitation and sedation scale with prolonged pain. Journal of perinatology : official journal of the California Perinatal Association 28:55-60

34. Holsti L, Grunau RE (2007) Initial validation of the Behavioral Indicators of Infant Pain (BIIP). Pain 132:264-272

35. Pölkki T, Korhonen A, Axelin A, Saarela T, Laukkala H (2014) Development and preliminary validation of the Neonatal Infant Acute Pain Assessment Scale (NIAPAS). International journal of nursing studies 51:1585-1594

36. Lawrence J, Alcock D, McGrath P, Kay J, MacMurray SB, Dulberg C (1993) The development of a tool to assess neonatal pain. Neonatal network : NN 12:59-66

37. Debillon T, Zupan V, Ravault N, Magny JF, Dehan M (2001) Development and initial validation of the EDIN scale, a new tool for assessing prolonged pain in preterm infants. Arch Dis Child Fetal Neonatal Ed 85:F36-41

38. Taddio A, Hogan ME, Moyer P, Girgis A, Gerges S, Wang L, Ipp M (2011) Evaluation of the reliability, validity and practicality of 3 measures of acute pain in infants undergoing immunization injections. Vaccine 29:1390-1394

39. Ramelet AS, Rees N, McDonald S, Bulsara M, Abu-Saad HH (2007) Development and preliminary psychometric testing of the Multidimensional Assessment of Pain Scale: MAPS. Paediatr Anaesth 17:333-340

40. Cignacco E, Mueller R, Hamers JP, Gessler P (2004) Pain assessment in the neonate using the Bernese Pain Scale for Neonates. Early Hum Dev 78:125-131

41. Büttner W, Finke W (2000) Analysis of behavioural and physiological parameters for the assessment of postoperative analgesic demand in newborns, infants and young children: a comprehensive report on seven consecutive studies. Paediatr Anaesth 10:303-318

42. O'Sullivan AT, Rowley S, Ellis S, Faasse K, Petrie KJ (2016) The Validity and Clinical Utility of the COVERS Scale and Pain Assessment Tool for Assessing Pain in Neonates Admitted to an Intensive Care Unit. Clin J Pain 32:51-57

43. Carbajal R, Paupe A, Hoenn E, Lenclen R, Olivier-Martin M (1997) APN: evaluation behavioral scale of acute pain in newborn infants. Archives de pediatrie : organe officiel de la Societe francaise de pediatrie 4:623-628

44. Milesi C, Cambonie G, Jacquot A, Barbotte E, Mesnage R, Masson F, Pidoux O, Ferragu F, Thevenot P, Mariette JB, Picaud JC (2010) Validation of a neonatal pain scale adapted to the new practices in caring for preterm newborns. Arch Dis Child Fetal Neonatal Ed 95:F263-266

45. Hodgkinson K, Bear M, Thorn J, Van Blaricum S (1994) Measuring pain in neonates: evaluating an instrument and developing a common language. The Australian journal of advanced nursing : a quarterly publication of the Royal Australian Nursing Federation 12:17-22

46. Hudson-Barr D, Capper-Michel B, Lambert S, Palermo TM, Morbeto K, Lombardo S (2002) Validation of the Pain Assessment in Neonates (PAIN) scale with the Neonatal Infant Pain Scale (NIPS). Neonatal network : NN 21:15-21

47. Schade JG, Joyce BA, Gerkensmeyer J, Keck JF (1996) Comparison of three preverbal scales for postoperative pain assessment in a diverse pediatric sample. J Pain Symptom Manage 12:348-359

48. Blauer T, Gerstmann D (1998) A simultaneous comparison of three neonatal pain scales during common NICU procedures. Clin J Pain 14:39-47

49. Soetenga D, Frank J, Pellino TA (1999) Assessment of the validity and reliability of the University of Wisconsin Children's Hospital Pain scale for Preverbal and Nonverbal Children. Pediatric nursing 25:670-676
