# Supplementary material for: Development of the neonatal pain response variable set: a mixed methods consensus process
Source: Eur J Pediatr. 2024 Jun 8;183(9):3719–26. doi: 10.1007/s00431-024-05559-7 (PMC11322254; doi:10.1007/s00431-024-05559-7)
Supplement: Supplementary file 2 — Supplementary file2 (DOCX 84 KB) [file 431_2024_5559_MOESM2_ESM.docx]

| **Table 1 Summary of expert' opinions to the panel meeting** | | | | |
| --- | --- | --- | --- | --- |
| **Type of variable** | **variable** | **Descriptor** | | **Experts' opinion** |
| **Contextual variable** | Hospital days | / | | / |
|  | Sleeping / Wake state | / | | Duplication with assessment indicators. **Recommended to deletion** |
|  | Mode of delivery | / | | / |
|  | Age | / | | / |
|  | Birth weight | / | | / |
|  | GA | / | | Duplication with assessment indicators. **Recommended to deletion** |
|  | Procedure Types | / | | / |
|  | Apgar (1 min) | / | | / |
|  | Apgar (5 min) | / | | / |
|  | Gender | / | | / |
|  | GA | / | | / |
|  | Nurse's perception | 1 | ● No Pain; ● Yes Pain. | / |

Continued

| **Physiological variable** | Requires O_2_ for  Sat > 95 | 1 | ⚫No Oxygen Required; | / |
| --- | --- | --- | --- | --- |
|  |  |  | ⚫ <30% Oxygen Required; |  |
|  |  |  | ⚫ >30% Oxygen Required |  |
|  | Oxygen requirement | 1 | ⚫ At baseline O_2_, Breathing comfortably; | / |
|  |  |  | ⚫ Requirement <30%, ↑ < 20%, Change in breathing pattern; |  |
|  |  |  | ⚫ Requirement >30%, ↑ > 20%, Significant change in breathing pattern. |  |
|  | SaO_2_ Changes | 1 | ⚫ Normal - no changes in additional oxygen need OR pre-emptive increase of max, 5 percentage points; | / |
|  |  |  | ⚫ Slight change - saturation level stays within set limits with oxygen increase of 6 ~10 percentage points; |  |
|  |  |  | ⚫ Clear change - saturation level decreases < 80 despite additional oxygen. |  |
|  | SpO_2_ Changes | 1 | ● Decrease ≤ 1.9; | The range of fluctuations is too small and less in line with clinical reality. **Recommended to use 3** |
|  |  |  | ● Decrease 2%~2.9%; |  |
|  |  |  | ● Decrease 3%~4.9%; |  |
|  |  |  | ● Decrease≥5%. |  |
|  |  | 2 | ● Normal; | Difficult to distinguish. **Not recommended** |
|  |  |  | ● Decrease with or without touch. |  |
|  |  | 3 | Decrease in Oxygen Saturation: | It should explicitly a change from the baseline value. **Accepted with modifications** |
|  |  |  | ● 0-2; |  |
|  |  |  | ● 3-5; |  |
|  |  |  | ● 6-8 or Increase in O_2_ |  |

Continued

| **Physiological variable** | Heart Rate Changes | 1 | ● 0~4; | It is not clear whether it is a comparison to the baseline value. **Not recommended** |
| --- | --- | --- | --- | --- |
|  |  |  | ● 5~14; |  |
|  |  |  | ● 15~24; |  |
|  |  |  | ● ＞25 |  |
|  |  | 2 | ● Normal: heart rate decreases/increases 0-5 beats from baseline | The heart rate of newborns can be affected by gestational age, and some newborns may have high or low heart rates. It is recommended that the specific range of heart rate be deleted and clarified to be a more meaningful value for the change from the baseline value. **Accepted with modifications** |
|  |  |  | ● Slight change: heart rate decreases/increases 6-20 beats from baseline OR heart rate is 170-189 beats/min; |  |
|  |  |  | ● Clear change: heart rate decreases/increases > 20 beats from baseline OR heart rate > 190 beats/min |  |
|  |  | 3 | ● Normal; | Lack of an assessment level for heart rate variability <20 beats and lack of definition of the length of observation time for "can be reduced to baseline levels", which is not operationalized well **Not recommended** |
|  |  |  | ● Increases ≥20 beats/min, could be reduced to baseline; |  |
|  |  |  | ● Increase ≥20 beats/min, do not decrease to baseline; |  |
|  |  |  | ● Increase ≥30 beats/min or bradycardia. |  |
|  |  | 4 | ● HR within 10% of baseline; | Need to calculate, poor operability. The use of specific beats changes is more intuitive. **Not recommended** |
|  |  |  | ● HR 11-20 % of baseline; |  |
|  |  |  | ● HR >20 % of baseline. |  |
|  |  | 5 | ● Depressions >15% below baseline; | Need to calculate, poor operability. The use of specific beats changes is more intuitive. **Not recommended** |
|  |  |  | ● Depressions to 15% below baseline; |  |
|  |  |  | ● Baseline; |  |
|  |  |  | ● Elevations to 15% above baseline; |  |
|  |  |  | ● Elevations >15% above baseline. |  |
|  |  | 6 | ● ＜10%; | Need to calculate, poor operability. The use of specific beats changes is more intuitive. **Not recommended** |
|  |  |  | ● ＞10%; |  |
|  |  |  | ● ＞50%. |  |

Continued

| **Physiological variable** | Breathing changes | 1 | ● Normal; | / |
| --- | --- | --- | --- | --- |
|  |  |  | ● Increase 10~14 times/min; |  |
|  |  |  | ● Increase 15~19 times/min; |  |
|  |  |  | ● Increase ≥20 times/min, tachypnea with thoracic contraction |  |
|  |  | 2 | ● Tachypnea at rest; | The scope of application is limited and can only be judged at rest. **Not recommended** |
|  |  |  | ● Apnea at rest or touch |  |
|  | Blood Pressure Changes | 1 | ● Normal | Measuring the blood pressure of the newborn frequently is easy to cause new stimulation and damage. **Recommended to delete** |
|  |  |  | ● Hypotensive/Hypertensive at rest |  |
|  |  | 2 | ● Depressions >15% below baseline; |  |
|  |  |  | ● Depressions to 15% below baseline; |  |
|  |  |  | ● Baseline; |  |
|  |  |  | ● Elevations to 15% above baseline; |  |
|  |  |  | ● Elevations >15% above baseline. |  |
|  | Color | 1 | ⚫ Pink; | / |
|  |  |  | ⚫ Flushed; |  |
|  |  |  | ⚫ Pale; |  |
|  |  |  | ⚫ Dusky. |  |
| **Behavioral variable** | Baseline Behavioral State | 1 | ⚫ Active and Awake; | / |
|  |  |  | ⚫ Quiet and Awake; |  |
|  |  |  | ⚫ Active and Asleep; |  |
|  |  |  | ⚫ Quiet and Asleep. |  |

Continued

| **Behavioral variable** | Sleep/Wake State | 1 | ● Sleeping/awake; | Status categorization is too simple and not clearly described. **Recommended to combined with 3** |
| --- | --- | --- | --- | --- |
|  |  |  | ● Fussy |  |
|  |  | 2 | ● No arousal to any stimuli, No spontaneous movement; | Only applicable in the state of sedation and analgesia. **Not recommended** |
|  |  |  | ● Arouses minimally to stimuli, Little spontaneous movement; |  |
|  |  |  | ● No sedation/No pain signs; |  |
|  |  |  | ● Restless, Squirming, Awakens frequently; |  |
|  |  |  | ● Arching, kicking, Constantly awake or Arouses minimally/no movement (not sedated) |  |
|  |  | 3 | ● Deep Sleep: Eyes closed, regular breathing, no movements of extremities; | Overly detailed descriptions.  **Recommended to combined with 1, modify to:** ● Deep Sleep; ● Active sleep; ●Active Awake;  ● Crying. |
|  |  |  | ● Active sleep: Eyes closed, twitches or startles of extremities, rapid eye movements, irregular breathing; |  |
|  |  |  | ● Drowsy: Eyes open (but roving or not focused) or closed, irregular breathing, some body movements; |  |
|  |  |  | ● Quite awake: Eyes open, focused, very few or no body movements; |  |
|  |  |  | ● Active awake: Eyes open, active extremity movements; |  |
|  |  |  | ● Agitated/crying: Upset, fussing, highly aroused, crying. |  |
|  |  | 4 | ● Sleeping most of time; | Requires longer observation and poor maneuverability. **Not recommended** |
|  |  |  | ● Wakes at frequent intervals-fussy; |  |
|  |  |  | ● Constantly awake (even when not disturbed). |  |
|  |  | 5 | ● Sleeping quietly with easy respirations; | Requires longer observation and poor maneuverability. **Not recommended** |
|  |  |  | ● Restless while asleep; |  |
|  |  |  | ● Sleeps intermittently; |  |
|  |  |  | ● Sleeping for prolonged periods of time interrupted by jerky movements or unable to sleep. |  |

Continued

| **Behavioral variable** | Sleep/Wake State | 6 | ● Calm or asleep; | It is only applicable to the observation of postoperative pain. **Not recommended** |
| --- | --- | --- | --- | --- |
|  |  |  | ● Hyperreactive: highly or excessively responsive or reactive to a nonpainful stimulus; |  |
|  |  |  | ● Shut down: decreased physical alertness and activity: does not communicate: or no eves contact |  |
|  | Alertness | 1 | ● Sleep or quite awake; | It is similar to the observation contents of the “sleep/awake state”. **Recommended to delete** |
|  |  |  | ● Drowsy |  |
|  |  |  | ● Active awake; |  |
|  |  |  | ● Highly aroused and agitated |  |
|  |  | 2 | ● Quiet sleep (eyes closed, no facial movement) |  |
|  |  |  | ● Active sleep (eyes closed, facial movement) |  |
|  |  |  | ● Quietly awake (eyes open, no facial movement) |  |
|  |  |  | ● Actively awake (eyes open, facial movement) |  |
|  |  |  | ● Awake and hyperalert |  |
|  |  | 3 | ● Calm/quiet (calm, quiet, asleep/awake); |  |
|  |  |  | ● Restless (restless and flailing a little/at times, can be calmed down); |  |
|  |  |  | ● Remarkably restless (restless and flailing nearly continuously, intermittent sleep) |  |
|  | Calmness / Agitation | 1 | ● Calm; | / |
|  |  |  | ● Slightly anxious; |  |
|  |  |  | ● Anxious; |  |
|  |  |  | ● Very anxious; |  |
|  |  |  | ● Panicky. |  |
|  | Sleep Pattern | 1 | ● Relaxed; | / |
|  |  |  | ● Agitated or withdrawn. |  |

Continued

| **Behavioral variable** | Quality of sleep | 1 | ● Continuously asleep; | / |
| --- | --- | --- | --- | --- |
|  |  |  | ● Awakened at frequent intervals; |  |
|  |  |  | ● Awake constantly. |  |
|  |  | 2 | ● Falls asleep easily; | Difficult to identify and poorly operationalized. **Not recommended** |
|  |  |  | ● Falls asleep with difficulty; |  |
|  |  |  | ● Frequent, spontaneous arousals, independent of nursing, restless sleep; |  |
|  |  |  | ● Sleepless. |  |
|  | Facial Action | 1 | ● Facial muscles fully relaxed, relaxed open mouth; | / |
|  |  |  | ● Normal facial tension; |  |
|  |  |  | ● Intermittent eye squeeze and brow furrow; |  |
|  |  |  | ● Continuous eye squeeze and brow furrow; |  |
|  |  |  | ● Facial muscles contorted and grimacing (eye squeeze, brow furrow, open mouth, nasal-labial lines) |  |
|  |  | 2 | ● Relaxed facial activity; | Difficulty in defining the length of time for "transient", "frequent", "continuous", "persistent". **Not recommended** |
|  |  |  | ● Transient grimaces with frowning, lip purse and chin quiver or tautness; |  |
|  |  |  | ● Frequent grimaces, lasting grimaces; |  |
|  |  |  | ● Permanent grimaces resembling crying or blank face. |  |
|  |  | 3 | ● Calm; | “Whining” and “duration” are hard to define. **Not recommended** |
|  |  |  | ● Whining or alternating closing and soft opening of eyes; |  |
|  |  |  | ● Eyelid contraction, frowning, or accentuation of the grooves, nasolabial furrow: |  |
|  |  |  | ● intermittent with return to calm(lightly); |  |
|  |  |  | ● the duration is slightly longer(moderately) |  |
|  |  |  | ● the duration is longer(severely) |  |

Continued

| **Behavioral variable** | Facial Action | 4 | ● Relaxed facial activity; | “Chin quiver” is affected by GA and development, it is not all newborns’ signs of pain. **Not recommended** |
| --- | --- | --- | --- | --- |
|  |  |  | ● Transient grimaces with frowning, lip purse and chin quiver or tautness; |  |
|  |  |  | ● Frequent grimaces, lasting grimaces; |  |
|  |  |  | ● Permanent grimaces resembling crying or blank face. |  |
|  |  | 5 | ● None/facial muscles relaxed; | The description of “Grimace” is not specific enough to judge clearly. **Not recommended** |
|  |  |  | ● Grimace, min-mod brow bulge, eye squeeze, nasolabial furrow; |  |
|  |  |  | ● Grimace/grunt, mod-max row bulge eye squeeze, nasolabial furrow |  |
|  |  | 6 | ● Totally relaxed, no tone or expression; | The description of “Grimace” is not specific enough to judge clearly; “Normal”, “Neutral”, “No tension” are hard to define. **Not recommended** |
|  |  |  | ● Reduced facial tone or expression; |  |
|  |  |  | ● Normal, neutral, no tension; |  |
|  |  |  | ● Increased tension, furrowed brow; |  |
|  |  |  | ● Contortion. grimace, vigorous cry. |  |
|  |  | 7 | ● Neutral/Smiling; | “Clenched teeth” is not suitable for newborns; “Full cry expression” is an indicator of crying and should not be used as an assessment of facial expression. **Not recommended** |
|  |  |  | ● Frowning/Grimacing; |  |
|  |  |  | ● Clenched teeth; |  |
|  |  |  | ● Full cry expression. |  |
|  |  | 8 | ● Smiling, calm, relaxed; | “Grimace” is not specific enough to judge clearly. It is difficult to distinguish between and (2), (3). **Not recommended** |
|  |  |  | ● Neutral expression, frowning, occasional grimace; |  |
|  |  |  | ● Occasional tense expression, slightly negative expression (e.g., grimace), brow bulge, shallow nasolabial furrow; (e.g., grimace), brow bulge, shallow nasolabial furrow; |  |
|  |  |  | ● Marked distress. Brow bulge, eyes squeezed shut, open mouth, taut tongue, deepening of nasolabial furrow. |  |

Continued

| **Behavioral variable** | Facial Action | 9 | ● Brow lowering; | The description is too detailed to operate; “Chin quiver” does not suitable to all newborns. **Not recommended** |
| --- | --- | --- | --- | --- |
|  |  |  | ● Eyes squeezed shut; |  |
|  |  |  | ● Deepening of the naso-labial furrow; |  |
|  |  |  | ● Open lips; |  |
|  |  |  | ● Vertical mouth stretch; |  |
|  |  |  | ● Horizontal mouth stretch; |  |
|  |  |  | ● Taut tongue; |  |
|  |  |  | ● Chin quiver; |  |
|  |  |  | ● Lip pursing; |  |
|  |  |  | ● Tongue protrusion. |  |
|  | Crying | 1 | ● No crying; | It is difficult to distinguish “Faint crying” from “Soft crying” and “Hard crying” from “Intense crying”. **Not recommended** |
|  |  |  | ● Faint crying; |  |
|  |  |  | ● Soft crying or moaning; |  |
|  |  |  | ● Hard crying; |  |
|  |  |  | ● Intense crying or screaming |  |
|  |  | 2 | ● No; | “Inconsolable” is similar to the observation contents of the “Consolability”. **Not recommended** |
|  |  |  | ● High pitched or visibly crying; |  |
|  |  |  | ● Inconsolable or difficult to soothe. |  |
|  |  | 3 | ● No; | The two states are too extreme, and the intermediate state needs to be supplemented. **Not recommended** |
|  |  |  | ● When disturbed, loud whimpering will not calm down after being soothed |  |
|  |  | 4 | ● Absent; | “Anxious” is a psychological emotional reaction, not applicable to newborns. **Not recommended** |
|  |  |  | ● Brief moaning, anxious; |  |
|  |  |  | ● Intermittent screaming; |  |
|  |  |  | ● Constant screaming. |  |

Continued

| **Behavioral variable** | Crying | 5 | ● No cry (quiet, not crying); | / |
| --- | --- | --- | --- | --- |
|  |  |  | ● Whimper (mild moaning, intermittent) |  |
|  |  |  | ● Vigorous crying (loud scream, shrill, continuous) |  |
|  | Duration of crying | 1 | ● No cry; | / |
|  |  |  | ● Crying less than 2 minutes; |  |
|  |  |  | ● Crying more than 2 minutes; |  |
|  |  |  | ● Shrill crying more than 2 min |  |
|  | Time to calm | 1 | ● No calm; | / |
|  |  |  | ● Calm less than 1 min; |  |
|  |  |  | ● More than 1 min; |  |
|  |  |  | ● More than 2 min. |  |
|  | Reacting to handling | 1 | ● Not sensitive to handling (tolerates handling/likes to be touched); | / |
|  |  |  | ● Painful/sensitive to handling (handling/ subdued, evasive movements, is disturbed by handling); |  |
|  |  |  | ● Extremely irritable/unresponsive (extremely irritable and sensitive to handling, does not tolerate handling/unresponsive) |  |
|  | Consolability | 1 | ● Quiet, total relaxation; | “Disconsolate” is difficult to judge in newborns. **Not recommended** |
|  |  |  | ● Calms down quickly in response to stroking or voice, or with sucking; |  |
|  |  |  | ● Calms down with difficulty; |  |
|  |  |  | ● Disconsolate. Sucks desperately. |  |
|  |  | 2 | ● Neutral; | The description is too brief. **Recommended to combined with 3** |
|  |  |  | ● Easy to console; |  |
|  |  |  | ● Not easy to console; |  |
|  |  |  | ● Inconsolable. |  |

Continued

| **Behavioral variable** | Consolability | 3 | ● Neutral, moves easily, interacts with people or environment, strong rhythmic suck on pacifier; | The description is too detailed to operate;  **Recommended to combined with 2, modify to**  ● Neutral: no crying, no need to console;  ● Easy to console: with holding, position change, or sucking; winces when touched/moved;  ● Not easy to console: followed by crying after consoling;  ● Inconsolable: continuous crying. |
| --- | --- | --- | --- | --- |
|  |  |  | ● Easy to console with holding, position change, or sucking; winces when touched/moved; |  |
|  |  |  | ● Consoles with moderate difficulty: sucks for very short periods, followed by crying; cries out when oved/touched; |  |
|  |  |  | ● Inconsolable: absent or disorganized sucking; high pitched cry or scream when touched or moved. |  |
|  |  | 4 | ● Smiles, attentive to voice; | “Smiles”, “Apprehension” and “Communicate” do not suitable to newborns. **Not recommended** |
|  |  |  | ● Transient apprehension during interactions with nurses; |  |
|  |  |  | ● Difficulty communicating with nurses. Cries in response to minor stimulation; |  |
|  |  |  | ● Refuses to communicate with nurses. No interpersonal rapport. Moans without stimulation. |  |
|  | Breathing pattern | 1 | ● No spontaneous respiration; | For children with mechanical ventilation only. **Not recommended** |
|  |  |  | ● spontaneous respiration on ventilator; |  |
|  |  |  | ● Unrest or resistance to ventilator; |  |
|  |  |  | ● Actively breathes against ventilator or coughs regularly; |  |
|  |  |  | ● Fights ventilator. |  |
|  |  | 2 | ● No spontaneous breathing; | For children with mechanical ventilation only. **Not recommended** |
|  |  |  | ● Shallow, intermittent breathing; |  |
|  |  |  | ● Quiel respiration, relaxed, usual pattern; |  |
|  |  |  | ● Increased rate and work of breathing. change from baseline; |  |
|  |  |  | ● Fights ventilator, coughs, chokes. |  |

Continued

| **Behavioral variable** | Breathing pattern | 3 | ● Relaxed (usual pattern for this infant) | / |
| --- | --- | --- | --- | --- |
|  |  |  | ● Change in breathing (irregular, faster than usual, gagging, breath holding) |  |
|  |  | 4 | ● No change; | “Respiratory distress” is not necessarily associated with pain response. **Not recommended** |
|  |  |  | ● Development or increase of respiratory distress; |  |
|  |  |  | ● Severe respiratory distress. |  |
|  |  | 5 | ● Adapted: the child is calm; | For children with mechanical ventilation only. **Not recommended** |
|  |  |  | ● Not adapted: breathing is uneven, draws breath/gasps between adjustments. |  |
|  | Body movements | 1 | ● No or minimal movement; | “Minimal” and “Vigorous” are affected by the health status of the newborn. **Recommended to modify to:**  ● No movement;  ● Up to three arm and/or leg movements;  ● More than three arm and/or leg movements. |
|  |  |  | ● Up to three slight arm and / or leg movements; |  |
|  |  |  | ● More than three slight arm and/or leg movements; |  |
|  |  |  | ● Up to three vigorous arm and/or leg movements; |  |
|  |  |  | ● More than three vigorous arm and/or leg movements, or whole body. |  |
|  |  | 2 | ● No movements or purposeful movements; | It is difficult to judge “Restless”, the definition is too vague. **Not recommended** |
|  |  |  | ● Restless; |  |
|  |  |  | ● Rigid and /or limited body movements. |  |
|  |  | 3 | ● Calm, Relaxed; | It is difficult to distinguish “Restless/Fidget”, “Moderate agitation or moderate mobility” and “Incessant agitation or strong voluntary irritability”. **Not recommended** |
|  |  |  | ● Restless/Fidget; |  |
|  |  |  | ● Moderate agitation or moderate mobility; |  |
|  |  |  | ● Incessant agitation or strong voluntary irritability. |  |

Continued

| **Behavioral variable** | Body movements | 4 | ● Relaxed body movements; | The observation content is too specific to operate; Fingers and toes can sometimes be constrained or protectively wrapped for reasons that cannot be observed. **Not recommended** |
| --- | --- | --- | --- | --- |
|  |  |  | ● Transient agitation, often quiet; |  |
|  |  |  | ● Frequent agitation but can be calmed down; |  |
|  |  |  | ● Permanent agitation with contraction of fingers and toes and hypertonia of limbs or infrequent, slow movements and prostration. |  |
|  |  | 5 | ● Calm or gentle movements; | The observation content is too specific to operate; Toes can sometimes be constrained or protectively wrapped for reasons that cannot be observed. **Not recommended** |
|  |  |  | ● Pedaling, toes spacing, stiff and elevated lower limbs, arm agitation, withdrawal: |  |
|  |  |  | ● Light, intermittent with return to calm; |  |
|  |  |  | ● Moderate; |  |
|  |  |  | ● very marked, permanent. |  |
|  |  | 6 | ● Relaxed/restrained; | The description is too brief. **Not recommended** |
|  |  |  | ● Flexed/extended. |  |
|  |  | 7 | ● Usual movements and activity, resting and relaxed; | It is difficult to observe “squirming” and “Agitation”. **Not recommended** |
|  |  |  | ● Partial movement (squirming arching, limb tensing clenching), attempt to avoid pain by withdrawing the limb where puncture is done. |  |
|  |  |  | ● Agitation with complex/generalized movements involving the head torso or other limbs, rigidity. |  |
|  |  | 8 | ● No movement or； | “Vigorous movements of head” is not suitable to newborns. **Not recommended** |
|  |  |  | ● Decreased activity, infrequent movements； |  |
|  |  |  | ● Occasional activity, usual movements; |  |
|  |  |  | ● Increased activity, flexion, and extension of extremities; |  |
|  |  |  | ● Vigorous movements of extremities, torso, head |  |
|  |  | 9 | ● Relaxed; | “Arching” is not suitable for newborns. **Not recommended** |
|  |  |  | ● Arms/legs flexed or extended, “time-out signals”; |  |
|  |  |  | ● Flailing, arching. |  |

Continued

| **Behavioral variable** | Body movements | 10 | ● Calm, slight; | It is difficult to observe. **Not recommended** |
| --- | --- | --- | --- | --- |
|  |  |  | ● Mild intermittent with return to calm; |  |
|  |  |  | ● Moderate; |  |
|  |  |  | ● Marked, continuous; |  |
|  |  |  | ● Global hypotonia. |  |
|  |  | 11 | ● Finger splay: hand suddenly open, with fingers straight and open; | “Clenching fist” is a basic phenomenon that occurs most of the time in newborns; Finger can sometimes be constrained or protectively wrapped for reasons that cannot be observed. **Not recommended** |
|  |  |  | ● Fisting: finger flexion, clenching fist |  |
|  |  | 12 | Lower limb posture: | The context of the assessment is more limited and better suited to targeting postoperative pain. **Not recommended** |
|  |  |  | ● Relaxed or straightened; |  |
|  |  |  | ● Kicking around; |  |
|  |  |  | ● Tightened in both legs. |  |
|  | Muscle tone | 1 | ● Muscles fully relaxed (open hands, dribbling, open mouth); | “Muscle tone” is susceptible to neonatal diseases and has poor applicability. **Recommended to Delete** |
|  |  |  | ● Reduced muscle tone; less resistance than normal; |  |
|  |  |  | ● Normal muscle tone; |  |
|  |  |  | ● Increased muscle tone (clenched hands and/or clenched, bent toes); |  |
|  |  |  | ● Extreme muscle tone (rigidity and flexion of fingers and/or toes). |  |
|  |  | 2 | ● Normal motor activity, baseline muscle tone; |  |
|  |  |  | ● Fidgeting; mild hypertonicity above baseline; |  |
|  |  |  | ● Moderate agitation or moderate immobility; intermittent flexion; moderate hypertonicity above baseline; |  |
|  |  |  | ● Thrashing, flailing incessant agitation or strong voluntary immobility; pronounced flexion; strong hypertonicity above baseline. |  |

Continued

| **Behavioral variable** | Muscle tone | 3 | ● No changes (Natural flexion or extension, slight movement of the limb); |  |
| --- | --- | --- | --- | --- |
|  |  |  | ● Changes (Stiffness/ relaxation of the touching limbs, increased/ disappeared resistance of passive motion joints) |  |
|  |  | 4 | ● No grasp reflex; Flaccid tone; |  |
|  |  |  | ● Weak grasp reflex; ↓ muscle tone; |  |
|  |  |  | ● No sedation/No pain signs; |  |
|  |  |  | ● Intermittent clenched toes, fist, or finger splay; Body is not tense; |  |
|  |  |  | ● Continuous clenched toes, fists, or finger splay; Body is tense. |  |
|  | Posture of the trunk | 1 | ● Relaxed; | According to the characteristics of clinical neonatal pain behavioral response: **Recommended to delete, use "Body movements".** |
|  |  |  | ● Basic relaxed, transient spasms; |  |
|  |  |  | ● Frequent spasm but can be relieved; |  |
|  |  |  | ● Continual spasm. |  |
|  |  | 2 | ● Middle; |  |
|  |  |  | ● Tossing and turning; |  |
|  |  |  | ● Buckling and stiffness. |  |
|  |  | 3 | ● Fingers stretched, shoulder lifted; |  |
|  |  |  | ● Bend and/or tense, fist clenched, trunk protected, limbs extended to midline, head and shoulders in a resistant position. |  |

| **Table 2 Summary of expert' opinions to the Delphi method** | | | | |  |
| --- | --- | --- | --- | --- | --- |
| **Type of variable** | **Number** | **Variable** | **Descriptor** | **Experts' opinion** | **Result** |
| **Contextual variable** | 1 | Hospital days | / | / | Retain |
|  | 2 | Mode of delivery | / | / | Retain |
|  | 3 | Age | / | / | Retain |
|  | 4 | Birth weight | / | / | Retain |
|  | 5 | Procedure Types | / | / | Retain |
|  | 6 | Gender | / | / | Retain |
|  | 7 | Apgar (1 min) | / | / | Retain |
|  | 8 | Apgar (5 min) | / | / | Retain |
|  | 9 | GA | / | / | Retain |
|  | 10 | Nurse's perception | ● No Pain | Overly subjective | Delete |
|  |  |  | ● Yes Pain |  |  |
| **Physiological variable** | 11 | Requires O_2_ for  Sat > 95 | ● No Oxygen Required; | In general, neonates have oxygen saturation levels of no more than 95 percent; For mechanically ventilated children only | Delete |
|  |  |  | ● <30% Oxygen Required; |  |  |
|  |  |  | ● >30% Oxygen Required. |  |  |
|  | 12 | Oxygen requirement | ● At baseline O_2_, Breathing comfortably; | Poor operability; Demand for oxygen does not directly reflect the existence of a relationship between it and pain | Delete |
|  |  |  | ● Requirement <30%, ↑ < 20%, Change in breathing pattern; |  |  |
|  |  |  | ● Requirement >30%, ↑ > 20%, Significant change in breathing pattern. |  |  |

Continued

| **Physiological variable** | 13 | SaO_2_ Changes | ● Normal - no changes in additional oxygen need OR pre-emptive increase of max, 5 percentage points; | The observation of this indicator relies on invasive means of monitoring such as blood gas analysis, which is poorly operable and can be harmful to the neonate, and it is recommended that it be replaced by SpO_2_ monitoring. | Delete |
| --- | --- | --- | --- | --- | --- |
|  |  |  | ● Slight change - saturation level stays within set limits with oxygen increase of 6 ~10 percentage points; |  |  |
|  |  |  | ● Clear change - saturation level decreases < 80 despite additional oxygen. |  |  |
|  | 14 | SpO_2_ Changes | Decrease in Oxygen Saturation: | / | Retain |
|  |  |  | ● 0-2; |  |  |
|  |  |  | ● 3-5; |  |  |
|  |  |  | ● 6-8 |  |  |
|  |  |  | ● >8 or increase in O_2_. |  |  |
|  | 15 | Heart Rate Changes | ● Normal: heart rate decreases/increases 0-5 beats from baseline; | / | Retain |
|  |  |  | ● Slight change: heart rate decreases/increases 6-20 beats from baseline; |  |  |
|  |  |  | ● Clear change: heart rate decreases/increases > 20 beats from baseline. |  |  |
|  | 16 | Breathing changes | ● Normal; | Respiratory rate of newborns is rarely monitored at all times in clinical practice and is poorly actionable; Newborns may experience apnea when stimulated by pain. | Delete |
|  |  |  | ● Increase 10~14 times/min; |  |  |
|  |  |  | ● Increase 15~19 times/min; |  |  |
|  |  |  | ● Increase ≥20 times/min, tachypnea with thoracic contraction. |  |  |

Continued

| **Physiological variable** | 17 | Color | ● Pink; | Difficult to judge in a short period of time. | Delete |
| --- | --- | --- | --- | --- | --- |
|  |  |  | ● Flushed; |  |  |
|  |  |  | ● Pale; |  |  |
|  |  |  | ● Dusky. |  |  |
| **Behavioral variable** | 18 | Baseline Behavioral State | ● Active and Awake; | / | Retain |
|  |  |  | ● Quiet and Awake; |  |  |
|  |  |  | ● Active and Asleep; |  |  |
|  |  |  | ● Quiet and Asleep. |  |  |
|  | 19 | Sleep/Wake State | ● Deep Sleep; | Poor operability; Confusion and duplication between its descriptors and the "Baseline Behavioral State"; Deep sleep and active are difficult to determine directly | Delete |
|  |  |  | ● Active sleep; |  |  |
|  |  |  | ● Active awake; |  |  |
|  |  |  | ● Crying. |  |  |
|  | 20 | Calmness/Agitation | ● Calm; | Need to determine "anxiety" and "panicky", which involves a psycho-emotional state and is not applicable to newborns. | Delete |
|  |  |  | ● Slightly anxious; |  |  |
|  |  |  | ● Anxious; |  |  |
|  |  |  | ● Very anxious; |  |  |
|  |  |  | ● Panicky. |  |  |
|  | 21 | Sleep Pattern | ● Relaxed; | Takes longer to observe, not easy to judge; Too many sleep disturbances in the NICU, poor maneuverability. | Delete |
|  |  |  | ● Agitated or withdrawn. |  |  |

Continued

| **Behavioral variable** | 22 | Quality of sleep | ● Continuously asleep; | Takes longer to observe, not easy to judge; Too many sleep disturbances in the NICU, poor maneuverability. | Delete |
| --- | --- | --- | --- | --- | --- |
|  |  |  | ● Awakened at frequent intervals; |  |  |
|  |  |  | ● Awake constantly. |  |  |
|  | 23 | Facial Action | ● facial muscles fully relaxed, relaxed open mouth; | / | Retain |
|  |  |  | ● normal facial tension; |  |  |
|  |  |  | ● intermittent eye squeeze and brow furrow; |  |  |
|  |  |  | ● continuous eye squeeze and brow furrow; |  |  |
|  |  |  | ● facial muscles contorted and grimacing (eye squeeze, brow furrow, open mouth, nasal-labial lines) |  |  |
|  | 24 | Crying | ● No cry (quiet, not crying); | / | Retain |
|  |  |  | ● Whimper (mild moaning, intermittent) |  |  |
|  |  |  | ● vigorous crying (loud scream, shrill, continuous) |  |  |
|  | 25 | Duration of crying | ● No cry; | The time point of observation needs to be defined, and it is suggested that it be modified to be the duration from the onset of crying to the cessation of crying after a painful stimulus | Retain |
|  |  |  | ● Crying less than 2 minutes; |  |  |
|  |  |  | ● Crying more than 2 minutes; |  |  |
|  |  |  | ● Shrill crying more than 2 min |  |  |
|  | 26 | Time to calm | ● No calm; | It takes long time to observe and the definition of calm is not specific and clear, making it difficult to judge | Delete |
|  |  |  | ● Calm less than 1 min; |  |  |
|  |  |  | ● More than 1 min; |  |  |
|  |  |  | ● More than 2 min. |  |  |

Continued

| **Behavioral variable** | 27 | Reacting to handling | ● Not sensitive to handling (tolerates handling/likes to be touched); | It is primarily judged by the neonate's limb movement and is recommended to be integrated with limb activity indicators. | Delete |
| --- | --- | --- | --- | --- | --- |
|  |  |  | ● Painful/sensitive to handling (handling/ subdued, evasive movements, is disturbed by handling); |  |  |
|  |  |  | ● Extremely irritable/unresponsive (extremely irritable and sensitive to handling, does not tolerate handling/unresponsive) |  |  |
|  | 28 | Consolability | ● Neutral: no crying, no need to console; | Each newborn's acceptance of the various types of soothing is inconsistent, and the length of soothing can have an impact on maneuverability. | Delete |
|  |  |  | ● Easy to console: withholding, position change, or sucking; |  |  |
|  |  |  | ● Not easy to console: followed by crying after consoling; |  |  |
|  |  |  | ● Inconsolable: continuous crying. |  |  |
|  | 29 | Breathing pattern | ● Relaxed (usual pattern for this infant) | / | Retain |
|  |  |  | ● Change in breathing (irregular, faster than usual, gagging, breath holding) |  |  |
|  | 30 | Body movements | ● No movement; | / | Retain |
|  |  |  | ● Up to three arm and/or leg movements; |  |  |
|  |  |  | ● More than three arm and/or leg movements. |  |  |
